# Search for Highly Divergent Tandem Repeats in Amino Acid Sequences

**DOI:** 10.3390/ijms22137096

**Published:** 2021-07-01

**Authors:** Valentina Rudenko, Eugene Korotkov

**Affiliations:** 1 Center of Bioengineering Research Center of Biotechnology RAS, 119071 Moscow, Russia; bioinf@rambler.ru; 2 Moscow Engineering Physics Institute, National Research Nuclear University MEPhI, 115409 Moscow, Russia

**Keywords:** tandem repeats, amino acid sequence, protein, mathematical method, cyclic alignment, pairwise correlation

## Abstract

We report a Method to Search for Highly Divergent Tandem Repeats (MSHDTR) in protein sequences which considers pairwise correlations between adjacent residues. MSHDTR was compared with some previously developed methods for searching for tandem repeats (TRs) in amino acid sequences, such as T-REKS and XSTREAM, which focus on the identification of TRs with significant sequence similarity, whereas MSHDTR detects repeats that significantly diverged during evolution, accumulating deletions, insertions, and substitutions. The application of MSHDTR to a search of the Swiss-Prot databank revealed over 15 thousand TR-containing amino acid sequences that were difficult to find using the other methods. Among the detected TRs, the most representative were those with consensus lengths of two and seven residues; these TRs were subjected to cluster analysis and the classes of patterns were identified. All TRs detected in this study have been combined into a databank accessible over the WWW.

## 1. Introduction

Protein molecules have a certain spatial structure, which is correlated with the functional role they play in the cell. Most proteins fold into a single specific conformation, which is characteristic for globular proteins composed of stable domains, whereas unstructured loop-forming fragments represent an insignificant part of the molecule. In contrast, non-globular proteins contain intrinsically disordered regions and low-complexity sequences. An important role in the formation of the spatial structure of the protein is played by repeats [1,2]. Repeats are quite common in amino acid sequences, and it has been reported that about 25% of proteins contain tandem repeats (TRs) [3]. The simplest and most common repeats form elements of the protein’s secondary structure. Thus, α-helices, which contain non-polar amino acids at each third or fourth position [4], have seven-residue long repeats with a consensus sequence axx–dxxx, where *a* and *d* are non-polar amino acids [5]. Structures such as 3_10_-helices have a periodicity of about 2.5 residues [4], whereas β-layers are characterized by alternation between polar and non-polar residues [6].

In most cases, there is an association between the repeat’s consensus length and the protein’s 3D structure, on which the most popular classification of repeat-containing proteins is based [7]. According to this, proteins with repeats fall into the following classes:

- crystalline (TR consensus length: two to three residues),
- fibrous (TR consensus length: three to four residues),
- solenoid (TR consensus length: 5–42 residues),
- proteins with domain-forming repeats (TR consensus length: ≥30 residues), including a subclass of β-propellers (TR consensus length: 44–60 residues).

The interest in TR-containing proteins is not accidental, as they play diverse roles in vital processes in the cell. Thus, proteins with repeats represent structural elements of cells and tissues (e.g., collagen and keratin) and provide a framework for protein–protein interactions [8] or, conversely, prevent cross-domain aggregation [9]. They may have catalytic [10] or inhibitory activity [11]. Furthermore, changes in the number of repetitive motifs and their consensuses can lead to genetic diseases [12,13,14]. Therefore, analysis of TRs in amino acid sequences should promote an understanding of protein structure and function. However, repetitive sequences in proteins evolve much faster than the other regions [15], and TRs may have weak identities owing to extensive evolutionary divergence, during which they accumulated insertions and deletions (indels) as well as substitutions of amino acids while preserving similar conformation and functional activity of the proteins. Therefore, the relatedness of individual repeats can be overlooked because of their significant sequence dissimilarity [16]. Although many methods and algorithms for detecting highly divergent TRs have been introduced, there is still no universal approach that can effectively identify TRs of an arbitrary length and degree of evolutionary separation.

A major effort has been put into the search for TRs using mathematical methods. Among these, statistical methods, although being the earliest, have not lost their relevance and are still quite popular [17]. They are based on observations of residue frequencies at a specific position in the consensus or the frequencies of occurrence of a substring in a given string. According to statistical data, a certain measure is formed that characterizes the collective similarity of repeats to each other. In earlier work [18], the Hamming distance was used as such a measure. It is noted that the algorithm works quite quickly, but it is suitable for finding only short repeats, with a consensus length of up to 10 amino acids and without indels.

The methods most frequently used to search for TRs are based on constructing sequence alignment with a determined repetitive consensus or self-alignment. The alignment method was one of the first applied to detect repeats in DNA sequences [19]. However, unlike DNA, amino acid sequences contain 20 characters which are, at the same time, much shorter and, thus, it is more difficult to search for statistically significant TRs. Therefore, prior to alignment, the amino acid residues are often combined into groups according to their physicochemical or spatial properties. For example, in the DAVROS algorithm, a weight matrix was calculated by a structural alignment builder (SAP) and used to find alignments between individual repeats [20], whereas in the Swelfe method, the angles between consecutive carbon atoms in the protein chain were denoted by specific symbols and used to re-code amino acid sequences prior to TR analysis [21].

Dynamic programming is used to find TRs with a consensus over 20 residues at the 3D level. The ProStrip program is based on translating protein backbone dihedral angles (calculated on the basis of four successive carbon atoms) into alpha characters for repeat detection [22]. The T-REKS method applies the k-means classification algorithm to accurately identify repeat lengths [23], and TRUST exploits information on the transitivity of individual repeats [24]. XSTREAM, based on the seed extension approach [25], is a very fast method that is suitable for full-scale scanning of amino acid sequence databanks [26]. Cyclic profile alignment, which uses previously defined periodic consensuses found by the information decomposition method, has made it possible to identify TRs in 94 protein families [27,28].

Since, from the mathematical point of view, TRs can be considered as periodicity in protein sequences, spectral approaches are also widely applied to search for repeats [29,30,31]. Spectral methods perform equally well for repeats of different lengths and degrees of diversity; however, they cannot detect periodicity if indels are present in individual periods [30]. An attempt was made to compensate for this drawback when the Fourier transform method was used to find repeats in membrane proteins: the hydrophobicity function of a protein fragment was averaged within a window of 9–11 residues, which was sliding along the sequence [31].

Hidden Markov Models (HMMs) such as HHrep [32] and HHRepID [33] are also used to find amino acid repeats. The idea is to build multiple alignments for the protein in question and use them to construct an HMM profile, which is then aligned with itself. Multiple alignments are obtained by iteratively applying PSI-BLAST; an alternative is to use multiple alignments constructed by other algorithms.

Recently, machine learning methods have become popular for big data analysis. These algorithms work by setting up a program for specific patterns and applying it to recognize a similar object in the analyzed sequence. For example, RAPHAEL software, which uses a geometric approach that simulates manual classification by an expert, can reveal spatial structures of the solenoid type, determine their periodicity, and assign indels [15]. Support vector machines are used for training. However, although machine learning methods are highly accurate, there is one significant drawback: they need prior information about the search object, such as the length and type of the repeat consensus or correct multiple alignment, which is not always available. Considering that the main goal is to identify TRs when the only input data are an amino acid sequence, machine learning methods can be of little help here.

Many programs for finding TRs are available as program codes or web servers, such as ProSTRIP [22], Swelfe [21], RAPHAEL [15], REPETITA [34], TRUST [24], and RADAR [26]. All these tools show high efficacy in identifying TRs of the known repeat families and accurately determine TR boundaries [15,22]; they work well if the TRs have accumulated an average number of substitutions per amino acid of the consensus sequence *S* < 0.5, but fail to detect repeats with a large number of residue substitutions and indels. However, it is the TRs with a high degree of evolutionary divergence that are of particular interest for studying the structural characteristics of protein molecules, as well as for further deciphering evolutionary processes.

Here, we describe a Method to Search for Highly Divergent TRs (MSHDTR), which can find repeats with *S* > 0.5 in the presence of indels. MSHDTR does not require any information other than the amino acid sequence. Using MSHDTR, we detected highly divergent TRs missed by the currently existing methods and constructed weight matrices characterizing the consensus sequence of repeats, which could be further used by other methods (for example, machine learning algorithms) to search for specific repeat domains in proteins.

## 2. Results

### 2.1. Determination of the Level of Statistical Significance Z_0_

MSHDTR was implemented in the Fortran programming language using the MPI parallel programming library. All calculations were performed in the computer cluster of the Research Center of Biotechnology RAS (Moscow, Russia).

First, we determined the statistical significance level *Z*_0_ using 132,133 sequences from the Swiss-Prot databank (release 2016_07; 551,987 sequences in total) denoted as the sequence set *Q*. The symbols in each sequence of the set *Q* were then randomly shuffled to obtain the set *Qr*; the two sets were searched for TRs with consensus lengths of 2–100 (the TR size was ≥14 residues, as recommended in [23]) and the false discovery rate (FDR) was calculated as *FP/(FP + TP)*, where *FP* and *TP* are false positive and true positive hits, respectively. The number of TRs in the set *Qr* is equal to *FP* and that in the set Q to *FP* + *TP*. For TRs with Z_0_ ≥ 6.0, *FP* = 1284 and *FP + TP* = 3720, which corresponds to the FDR of 34.5%; this FDR value was chosen because it is typical for most methods used to detect TRs in proteins (see the section below).

The search for TRs in the Swiss-Prot databank produced 15,035 amino acid sequences that contained TRs with different consensus lengths. Considering that one sequence may have several non-overlapping TRs, the total number of identified regions with TRs was 15,407.

### 2.2. Comparison of MSHDTR with Other Methods of TR Detection

To correctly compare the performance of MSHDTR with those of the T-REKS [23] and XSTREAM [25] algorithms, we verified the similarity of their FDRs, which were calculated using the sets *Q* and *Qr* as described in Section 2.1. The FDRs for T-REKS with *psim* = 0.75 and XSTREAM with the default parameters were 33.5% and 31.3%, respectively, and could be considered similar to that of MSHDTR (34.5%). In further analysis, we used T-REKS with *psim* = 0.75, XSTREAM with default parameters, and MSHDTR with *Z*_0_ ≥ 6.0.

Next, MSHDTR, T-REKS, and XSTREAM were applied to various artificial sequences with TRs. For this, we generated random sets of amino acid sequences, which contained different numbers of repeats with a length *n* (2, 5, 7, 10, 20, 40, 60, 80, and 100 residues); homo-repeats were excluded from consideration. Random fragments of length *n* were created based on the average residue composition according to Swiss-Prot; for each *n*, 100 different random chunks were obtained, and each fragment was then repeated *k* times (*k* = 4, 8, 16, 24, 32, and 50). As a result, we generated an amino acid sequence of length *nk* that contained TRs of a consensus length *n*.

A total set of 5400 sequences, denoted as *W*(0), was used to perform the TR search methods. In each sequence of set *W*(0), we introduced random indels at the rate of 1 per 100 residues and substitutions of a certain percent *i* (25%, 50%, 75%, 90%, 100%, 110%, 120%, and 150%) and created the sets *W*(*i*). Each sequence from the sets *W*(*i*) was inserted into a random position of a random sequence consisting of 600 residues, and the information was stored to test the accuracy of TR boundary determination. TR identification in the sets *W*(*i*) was considered to be correct if the consensus length of the found repeat exactly coincided with *n*, and the TR-containing region overlapped with the artificial repeat region of length *nk* by at least 50%. If several non-overlapping regions with the same *n* were found inside the tested TRs, their lengths were summed during calculation of the intersection length.

The results of TR identification in the sets *W*(*i*) by T-REKS, XSTREAM, and MSHDTR are shown in Table 1. All three methods revealed a relatively high percentage of TRs in weakly divergent sequences. However, with the increase in mutation frequency, the recognition efficiency of T-REKS and XSTREAM rapidly decreased and they could not detect TRs in sequences with ≥50% random substitutions, whereas MSHDTR was able to identify 17.5% of TRs with the divergence rate of 100%. At the same time, MSHDTR missed some TRs; one possible reason was that it recognized highly divergent TRs when *nk* was large enough and, consequently, overlooked short regions such as two-residue fragments repeated four times, which were successfully identified by T-REKS. Therefore, the next task of the study was to determine the application area of the developed method, i.e., the conditions (*nk* and degree of evolutionary divergence) under which MSHDTR could reliably identify TRs.

### 2.3. Determination of the Optimal Application Scope for MSHDTR

To determine the scope of the MSHDTR application, we calculated the divergence degree of TRs found in Swiss-Prot using the following algorithm.

1. We let *k* be the number of TRs with a length *n* and built multiple alignments for the TRs found. Columns with the number of residues less than *k*/2 were disregarded.

2. The coincidence matrix *M*_2_(*i, j*)*,* where *i* = 1, 2, …, 20 and *j* = 1…*n*_1_, was calculated. The length of the multiple alignment was *n*_1_ ≥ *n*; *m*_2_(*i*, *j*) was an element of the matrix *M*_2_(*i, j*) filled according to the multiple alignment and was equal to the number of amino acids of type *i* in column *j* of the multiple alignment.

3. For TRs, we calculated the consensus sequence. The consensus sequence *s*_3_(*j*) (*j* = 1,2, …, *n*_1_), where *i* was in position *j* if *m*_2_(*i, j*) was the maximum for *i* = 1, 2, …, 20.

4. Finally, we calculated *v*, which was the number of residues in all repeats of the multiple alignment that coincided with the respective position of the consensus sequence. The total number of residues *v*_1_ was also determined as the sum of all elements of the matrix *M*_2_(*i, j*). Then, the degree of divergence was determined as follows:

(1) $$S=1-\frac{v}{v_{1}}$$

**Figure 1:**
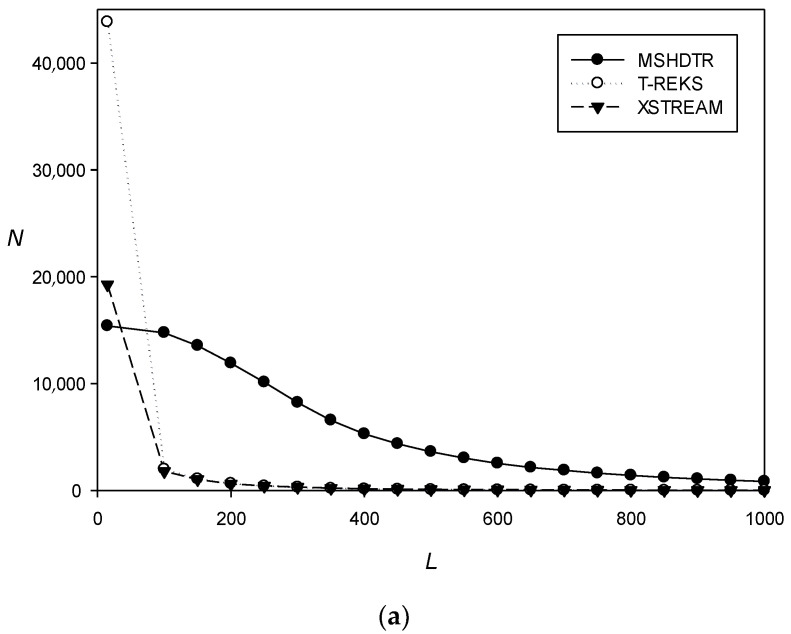

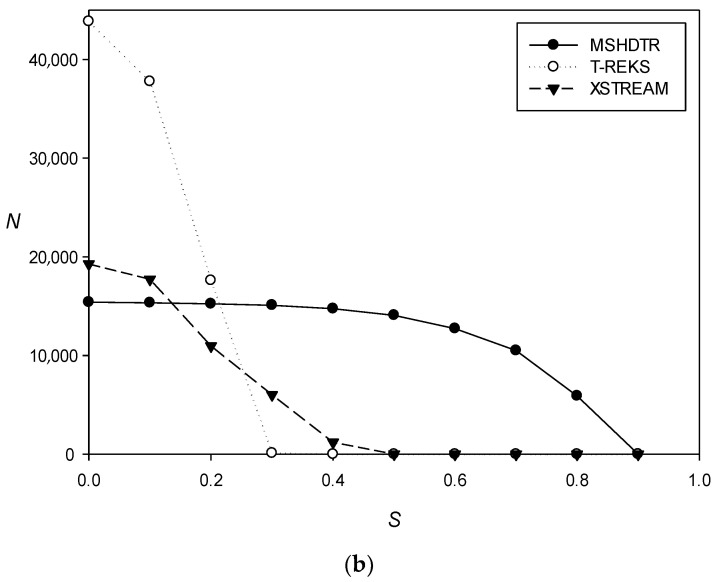
The number of TRs (*N*) found in Swiss-Prot by different methods depending on (**a**) the TR length (*L*) and (**b**) the degree of TR divergence (*S*).

MSHDTR, T-REKS, and XSTREAM were used to search for TRs in Swiss-Prot, and *S* for the found TRs was calculated using Equation (1). The results indicated that T-REKS and XSTREAM could identify more TRs than MSHDTR: 41,375 and 19,255 vs. 15,407, respectively. However, it should be noted that the TRs detected by MSHDTR were very different from those found by the other methods. In particular, we examined properties such as the degree of divergence (*S*) and the length of the sequence fragment (*L*) where TRs were found. Figure 1a shows the dependence of the number of TR-containing regions (*N*) on the length *L* The function value represents the absolute number of regions whose lengths was greater or equal to *L*. It can be seen from the graph that the lengths of most such regions detected by T-REKS and XSTREAM did not exceed 100 residues (31 and 47, respectively), whereas those identified by MSHDTR were much longer: 399 residues.

Figure 1b shows the dependence of the number of TR-containing regions (*N*) on the degree of divergence *S* Equation (1). The value of the function shows the number of regions with TRs that had a divergence degree greater or equal to the argument. Although T-REKS detected almost 2.5 times more TRs than the other two methods, most of them were short, practically identical repeats. Typically, T-REKS and XSTREAM identified TRs with a high degree of similarity, whereas MSHDTR recognized repeats with significant evolutionary divergence.

Table 2 shows the number of TRs found in Swiss-Prot by the three methods with and without filtering according to the *S* value; it is evident that at *S* > 0.5, only MSHDTR could detect TRs.

We also compared MSHDTR with a mathematical approach that implements hidden Markov models [33]. The HHRepID software was obtained from the server [35]. For testing, we created artificial repeats (*SEQ0–SEQ7*) with different percentages of random substitutions (25%, 50%, 75%, 90%, 100%, 110%, 120%, or 150%). TRs were constructed from 100 repeats with a consensus of seven residues and inserted into random positions of random amino acid sequences 600 residues long. The positions of the artificial TRs in random sequences are shown in Table 3 (*l*_0_, *r*_0_).

The identification was considered correct if the overlap with TRs was at least 50% and the consensus length of the repeat differed from 7 by no more than 1 or a multiple of 7. The results shown in Table 3 indicated that MSHDTR confidently identified the artificial TRs (*Z* > 10.0) with a degree of evolutionary divergence of up to 120%; over that (150%), the presence of TRs could hardly be detected (*Z* = 5.0). In all cases, the identified areas included complete TRs. It is important to note that the found consensuses exactly corresponded to the TR consensus length in the artificial sequence.

In contrast, HHRepID correctly detected only two sequences with evolutionary divergence rates of 25% and 50%. In *SEQ1*, HHRepID identified two distinct regions with a consensus length of seven residues, which were included in real TRs. At higher levels of evolutionary divergence (75% and 90%), only short fragments with the required or multiple consensus lengths were detected, and the overlap with the real TRs was less than 33% of the length. At the mutation level of 100%, only short parts of sequences with repeats of 14 and 15 residues were found.

The comparison of MSHDTR and HHRepID revealed not only that MSHDTR was able to recognize highly divergent TRs but also that it could more accurately detect the boundaries of TR fragments and the consensus length. At the same time, HHRepID tended to overestimate the consensus length, which was either presented as a multiple of the real length or differed from it by several residues; furthermore, it frequently significantly underestimated the length of the region occupied by TRs.

### 2.4. Examination of TRs Detected in Swiss-Prot

#### 2.4.1. TR Statistics by Period Length

Next, we examined the distribution of TRs over the consensus length *n*. It turned out that short TRs with consensus lengths from 2 to 11 amino acids were the most frequently observed in proteins, accounting for 71.0% of all TRs found using MSHDTR (Table 4), which can be associated with the presence of secondary protein structures such as β-sheets and α-helices. Along with short repeats, our method could detect longer TRs, which are apparently responsible for more complex spatial structures [7].

#### 2.4.2. Classification of TRs with a Length n Equal to Two and Seven Residues

The results shown in Table 5 indicated that TRs with consensus lengths of two and seven amino acids were the most common; the former are likely to be associated with β-sheets, as 2 is close to 2.3 residues, corresponding to a typical period for β-structures [4], whereas the latter are known to be located on α-helices, in which each turn consists of 3.6 residues [36].

**Figure 2:**
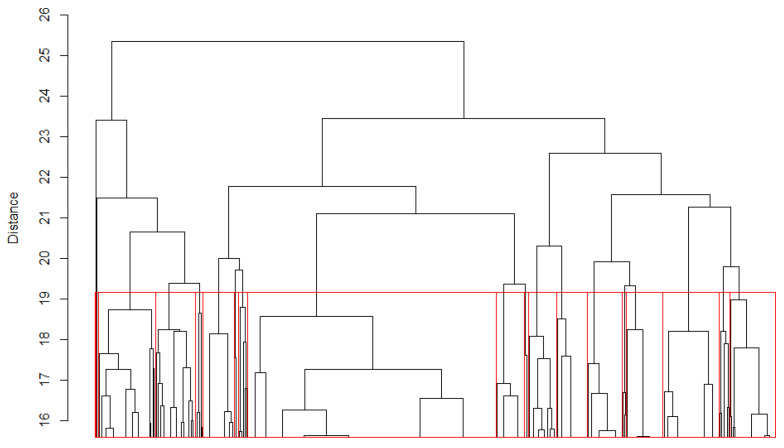
Cluster dendrogram of 2104 TRs with the consensus length of two residues. Red vertical lines indicate the division into classes.

**Figure 3:**
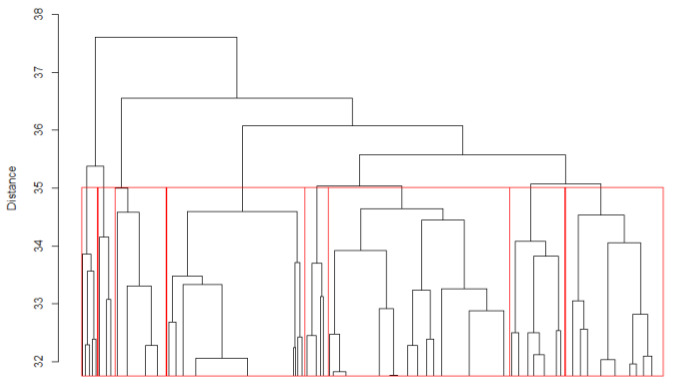
Cluster dendrogram of 1650 TRs with the consensus length of seven residues. Red vertical lines indicate the division into classes.

We classified TRs with the consensuses of two and seven residues, as described in Section 4.4 of the Materials and Methods. Figure 2 and Figure 3 show the clustering dendrograms for TRs with the lengths of two and seven residues, respectively. Only the upper parts of the dendrograms are shown, since the total number of objects was too large for display.

We calculated distance *B*_0_ at which the grouping into classes was not random (Equation (8)). The matrices *M_max_(n*, 25) (*n* = 2 or 7) were obtained by analysis of the Swiss-Prot databank; in total, 2104 *M_max_*(2, 25) and 1650 *M_max_*(7, 25) were obtained. Next, we created two sets, *MR*_2_ and *MR*_7_, which contained 2104 2 × 25 matrices and 1650 7 × 25 matrices (2 and 7 are the number of rows and 25 is the number of columns in the matrix). Each *i*-th matrix in the sets *MR*_2_ and *MR*_7_ was obtained by randomly rearranging the elements of matrix *i* from the original set *M_max_(n*, 25). We then performed cluster analysis for each set *MR*_2_ and *MR*_7_ and determined the average number of elements in the class for different values of the distance *B*, denoted as *Y_n_(B)* (where *n* is equal to 2 or 7). We calculated the mathematical expectation and variance of the random variable *Y_n_(B)* to classify the sets *MR*_2_ and *MR*_7_, and determined the average number of elements in the class *X_n_(B)* for the real matrices *M_max_(n*, 25). After that, we calculated the value of *Z_n_(B)* [37]:

(2) $$Z_{n}(B)=\frac{X_{n}(B)-Y_{n}(B)}{\sqrt{D(X_{n}(B))/r_{1}+D(Y_{n}(B))/r_{2}}}$$

where *r*_1_ and *r*_2_ are the numbers of classes obtained by classifying the matrices *M_max_(n*, 25) and the set *MR_n_*, respectively, at the level *B*.

We chose *B*_0_ = 19 for *n* = 2 and *B*_0_ = 35 for *n* = 7; in both cases, *Z_n_(B)* exceeded 10. The obtained classes at the given levels are framed in red in the dendrograms (Figure 2 and Figure 3). For *n* = 2 residues, there were 19 classes; among these, 11 were the most representative, containing over 90% of the elements. For *n* = 7 residues, the number of classes at the level *B*_0_ = 35 was equal to 8.

For each created class, we determined the common class matrices *M_max_(n*, 25). The *(i, j)* elements of the matrix were found by averaging them in all matrices included in the class, taking the phase into account. To do this, we first found the central matrix for each class, from which the total distance to the rest of the class elements was minimal. The order of the rows in the other matrices of the class was shifted before averaging to the phase with the central matrix of the class by cyclic permutation of the rows. The phase for the matrix was considered to be correct if the distance *B* between the matrix and the central matrix was minimal. Class matrices for *n* = 2 or 7 residues are provided in the Supplemental Materials.

### 2.5. Performance of MSHDTR in Identifying Weakly Similar TRs from Swiss-Prot

A specific feature of our method is that it is able to detect TRs in rather extended protein regions as well as highly divergent TRs with *S* > 0.5. MSHDTR can also successfully detect TRs with a very high *Z* (>30.0), such as those present in collagen-like proteins, serine-aspartate repeat proteins, and zinc finger proteins; these proteins have been previously reported as TR-containing and were confirmed as such here by using MSHDTR. However, the performance of MSHDTR is more notable in the analysis of sequences with a low *Z* in the interval of 8.0–10.0, in which most TRs are not recognized by other methods. Below, we present two examples of such sequences from Swiss-Prot.

1. Hexokinase-1 from *Mus musculus* (P17710; sequence length: 974 amino acids). The enzyme is the first in the glycolysis pathway, where it catalyzes the phosphorylation of hexoses; it consists of two sub-units and is ubiquitously expressed in all mammalian tissues. MSHDTR could detect TRs of *n* = 64, *S* = 0.75, and *Z* = 9.0 at positions from 96 to 805. Table 5 shows the multiple alignment of the detected repeats presented using the five-character code (Materials and Methods, Section 4.1).

Repeats are shown using the five-character code introduced in Section 4.1 (K, N, I, M, and T indicate non-polar, polar, aromatic, positively charged, and negatively charged residues, respectively).

**Figure 4:**
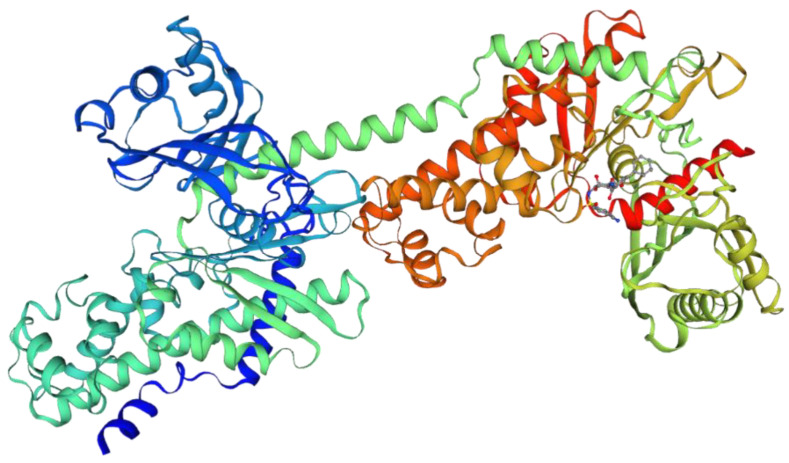
3D structure of the 73–969 residue region of the *Mus musculus* hexokinase-1 monomer.

The TRs identified by MSHDTR have not been previously detected by other methods and are not described in Swiss-Prot. The 3D structure of the enzyme monomer 73–969 is shown in Figure 4. The molecule consists of alternating helical and non-helical regions, which are included in the 64-residue consensus of the found TRs.

**Figure 5:**
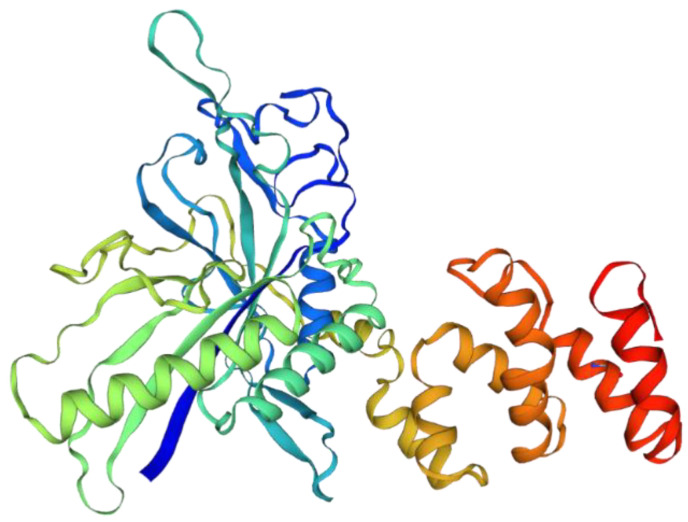
3D structure of the 3–403 residue region of the aspartyl/glutamyl-tRNA (Asn/Gln) amidotransferase subunit B monomer.

2. Aspartyl/glutamyl-tRNA (Asn/Gln) amidotransferase subunit B from *Pseudomonas aeruginosa* strain UCBPP-PA14 (Q02GV7; sequence length: 481 amino acids). MSHDTR could detect TRs six residues long (*S* = 0.81, *Z* = 7.9) at positions from 186 to 469. The multiple alignment of the found TRs is shown in Table 6 and the 3D structure of the enzyme monomer (3–403 residues) is shown in Figure 5.

Repeats are shown using the five-character code introduced in Section 4.1 (K, N, I, M, and T indicate non-polar, polar, aromatic, positively charged, and negatively charged residues, respectively).

The 15,407 TRs found by our program in Swiss-Prot have been placed in a database (http://victoria.biengi.ac.ru/aarep/ access date: 30 June 2021) running under MySQL DBMS. All TRs in the database are presented in the form of a list; detailed information on specific features such as the matrix *M_max_(n*, 25) and alignments can be accessed through the hyperlink. There is also a standard filtering functionality for finding TRs with the desired parameters, such as a specified range of consensus lengths or a *Z* value.

## 3. Discussion

Here, we described a method that can detect TRs with weak similarity in protein sequences. MSHDTR applied to the search of the Swiss-Prot databank, comprising over 500 thousand sequences, revealed more than 15 thousand proteins with TRs, including about 14 thousand containing highly divergent TRs with *S* > 0.5, which constituted 2.8% of the whole databank.

The ability of MSHDTR to identify TRs with *S* > 0.5 can be due to two factors. First, we grouped amino acids according to their physicochemical properties (non-polar, polar, aromatic, positively charged, and negatively charged) and re-coded the protein sequences using five instead of 20 symbols. As a result, MSHDTR considers the positions of TRs containing amino acids of the same group as identical; therefore, TRs for which the probability of residue substitutions within a group was higher than that between groups detected as similar by this method, which would not happen in case of 20-letter sequences.

Second, in the search for TRs, MSHDTR considers the correlation of neighboring residues. The weighting matrix for alignment was calculated based on the frequencies of pairs of symbols that encoded groups of amino acids rather than individual residues and, thus, reflected correlations between adjacent amino acids in TRs. As a result, MSHDTR could recognize highly divergent TRs that otherwise would not be possible to detect at a statistically significant level using previously developed methods.

At the same time, MSHDTR skips TRs present in small numbers, which limits its applicability. Thus, the scopes of the existing methods, such as T-REKS and XSTREAM, and our method regarding TR detection are fundamentally different. If the aim is to find reasonably well-preserved TRs (*S* < 0.5) with a small number of copies, then T-REKS and XSTREAM should be chosen. If, however, the aim is to detect highly divergent TRs (*S* > 0.5) present in large numbers, MSHDTR should be applied. Therefore, to cover the whole spectrum of potentially existing TRs, we recommend using T-REKS and XSTREAM in conjunction with MSHDTR.

We also compared the performance of MSHDTR with that of the Fourier transform applied using the procedure described previously [38]. It appeared that the Fourier transform could detect only 11% of the TR-containing sequences among those identified by MSHDTR (1707 out of 15,407). While comparing the two approaches, we assumed that the consensus length *n* of TRs detected by MSHDTR and that defined by the Fourier approach should differ by ≤10%. Most overlaps were observed for *n* < 10 residues, and only less than 10% of the 1707 sequences detected by the Fourier transform had a period length of >10 residues.

The class matrices created here for *n* = 2 and *n* = 7 can be used to search for divergent repeats belonging to these classes. These matrices should make it possible to identify extremely dissimilar TRs of a specific type that could not be detected before and to do it more quickly, since there would be no need to pre-generate the weight matrices used in the dynamic programming method.

Highly divergent TRs imply some regularity, which is reflected in the spatial organization of the protein molecule. An extremely interesting question is whether TRs are found in intrinsically disordered proteins and regions (IDP, IDR).

It is well known that IDPs do not have unique spatial structures. Their structure and functions are customizable to interact with different partners using alternative splicing and post-translational modifications. Thus, they contribute to increasing the complexity of biological organization [39,40]. The amount of IDPs is strictly controlled at the cellular level. IDP regulation faults are associated with diseases such as cancer, diabetes, and cardiovascular disease [41]. It is assumed that more than half of eukaryotic transcription factors contain IDRs [42].

Previously, the DisProt database containing IDPs was created [43]. We analyzed sequences from DisProt and found some IDRs containing TRs, for example, protein P38398 from *Homo sapiens*, Breast cancer Type 1 susceptibility protein (length: 1863 residues). It has 83.2% disordered content from 100 to 1649 residues. At the same time, in it, we detected TRs from 31 to 1768 residues with a consensus length of 24, *Z* = 10.7, and *S* = 0.81. We can also consider protein P30185 from *Arabidopsis thaliana*, Dehydrin Rab18 (length: 186 residues). The disordered content was 100%. It has TRs with a consensus length of seven residues, *Z* = 7.2, and *S* = 0.61 from 10 to 180 residues.

Thus the developed method detects such divergent patterns thatallows the protein to accept the desired conformation only in certain interactions.

## 4. Materials and Methods

### 4.1. Re-Coding of Amino Acid Sequences According to Side-Chain Polarity

Before the search for TRs, we divided the 20 amino acids into 5 groups according to the polarity of their side chains—non-polar, polar, aromatic, positively charged, and negatively charged—and assigned a specific letter to each group: *K* (G, A, V, I, L, P), *N* (S, T, C, M, Q, N), *I* (F, Y, W), *M* (K, R, H), and *T* (D, E), respectively [44]. Next, each amino acid sequence in the Swiss-Prot databank was re-coded using the 5 symbols. The aim of such re-coding was to take the correlation of adjacent residues into account, which was not feasible with the 20-letter code because of the large number of possible amino acid pairs. The resulting symbolic sequence was designated as *Seq(l)*, *l* = 1, 2, …, *NN* (where *NN* is the volume of the Swiss-Prot databank) and was searched for TRs using the algorithm MSHDTR.

### 4.2. Cyclic Alignment Search Method Considering Pairwise Correlations

To find TRs in *Seq(l)*, we used a mathematical algorithm that included 4 steps. First, we created a set of random position–weight matrices (PWMs) *Q(n)* of size (*n*, 25), where *n* is the consensus length of TRs. Set *Q(n)* contained 10^3^ matrices *M(n*, 25), where *n* and 25 are the numbers of rows and columns, respectively, in each PWM, since we considered correlations of adjacent symbols in *Seq(l)*.

Second, we used an iterative procedure to determine the matrix *M_max_*(*n*, 25) that best described TRs of length *n* present in the sequence *Seq(l)*. For this, we performed local alignment of the sequences *S*_1_ and *Seq(l)* using the matrix *M*(*n*, 25) from the set *Q(n)* [45]. The sequence *S*_1_*(n)* was created artificially and contained a fragment 1, 2, …, *n*, which was repeated in tandem over the length *L*. The matrix *M_max_*(*n*, 25) was considered to have the best local alignment with the sequence *Seq(l)* if it had the highest value of the similarity function *F_max_(n)*.

In the third step, this procedure was iterated for a consensus length *n* from 2 to 100, and *M_max_*(*n*, 25) and *F_max_(n)* were determined for each length *n*.

Finally, we evaluated the statistical significance of TRs with consensus lengths *n* = 2–100 using the Monte Carlo method. Each step of the developed algorithm is described in detail below.

#### 4.2.1. Creation of a Set of Random Matrices Q(n)

We generated the sequence *S*_1_(*n*) that contained the fragment 1, 2, …, *n* repeated 1000 times; thus, the sequence *S*_1_(*n*) had a length *L* = 1000 *n*. Next, we created the sequence *S*_2_ of length *L*, where numbers from 1 to 5 occurred in a random order and with equal probabilities. Next, we filled in the matrix *M*_1_*(n*, 25) using an iterative procedure. Initially, the elements of the matrix were zeroed; in this case, *M*_1_(*s*_1_(*i*)*, k*) *= M*_1_(*s*_1_(*i*)*, k*) + 1, where *i* = 2 to *L*, *s*_1_(*i*) is the *i*-th element of the sequence *S*_1_(*n*), and *k = s*_2_(*i* − 1) + 5 *(s*_2_(*i*) *−* 1). The sum of the elements of the matrix *M*_1_(*n*, 25) is equal to *L*-1. Based on the matrix *M*_1_(*n*, 25), we calculated PWM *M(n*, 25) using the formula:

(3) $$M\left(i,j\right)=\frac{M_{1}\left(i,j\right)-(L-1)p(i,j)}{\sqrt{\left(L-1)p(i,j)(1-p(i,j)\right)}}$$

where $p\left(i,j\right)=x\left(i\right)y\left(j\right)/{\left(L-1\right)}^{2}$, $x(i)=\sum_{j=1}^{25}M_{1}(i,j)$, and $y(j)=\sum_{i=1}^{n}M_{1}(i,j)$.

Thus, we created the first *M*(*n*, 25) from the set *Q*(*n*). After that, we randomly mixed the numbers in the sequence *S*_2_, re-filled matrix *M*_1_(*n*, 25), and calculated the second matrix *M(n*, 25) by Equation (3). In total, we created 10^3^ matrices *M(n*, 25) for each *Q(n)*, where *n* varied from 2 to 100.

#### 4.2.2. Iterative Procedure for Finding the Matrix M_max_(n, 25)

The sets *Q*(*n*) created in Section 4.2.1 were used to calculate the matrix *M_max_(n*, 25*)* according to the following algorithm:

1. Matrix transformation

We took the first matrix *M(n*, 25) from the set *Q*(*n*) and calculated:

(4) $$R^{2}=\sum_{i=1}^{n}\sum_{j=1}^{25}m{\left(i,j\right)}^{2}$$

(5) $$K_{d}=\sum_{i=1}^{n}\sum_{j=1}^{25}m(i,j)p_{1}(i)p_{2}(j)$$

where *p*_1_ (*i*) is the probability of symbols in *S*_1_, which is equal to 1/*n* for any *i*, and *p*_1_(*k*) and *p*_2_(*l*) are the probabilities of the numbers *k* and *l*, respectively, in *S*_2_ (*k, l* = {1, 2, 3, 4, 5}).

To make each matrix from the set *Q*(*n*) have the same *R^2^* and *K_d_*, we performed matrix transformation as described in detail in [46], after which, all the matrices had *R*_0_ *=* 110 *n* and *K_d_* = −1.0; the transformed matrix was denoted as *MT(n,* 25*)*. The aim of the transformation was to obtain approximately the same distribution for the similarity function *F*(*n*) [46] to analyze random sequences, so that the local alignment with the highest *F_max_*(*n*) could be selected as the most statistically significant. Such an assumption greatly speeds up calculations.

2. Local Alignment of the Sequences *S*_1_*(n*) and *Seq(l*)

Let the length of the sequence *Seq(l)* be *L*. To calculate the local alignment, we used the sequence *S*_1_(*n*) introduced in Section 4.2.1, whose length was equal to that of the sequence *Seq*(*l*). For local alignment of the sequences *S*_1_(*n*) and *Seq*(*l*), we calculated the matrix of similarity function *F* using the formula:

(6) $$F(i,j)=\max\left\{\begin{array}{l} 0\\ F(i-1,j-1)+MT(s_{1}(j),t)\\ F(i,j-1)-del\\ F(i-1,j)-del \end{array}\right\}$$

where *t* = *s*(*k*) + 5(*s*(*i*) − 1)); *i* and *j* range from 2 to *L*; *s(i)* and *s*_1_(*j*) are elements of the sequences *S*(*l*) and *S*_1_, respectively; and *t* indicates the fact that in the matrix *MT*, symbol pairs are considered.

For calculations using Equation (6), we need to find the previous position *k* already included in the alignment. The process of searching for *k* and filling the matrix *F* with the dimensions *(L, L)* is described in [47] in their Section 2.3.

We took *F*(*i*, 0) = 0 and *F*(0, *j*) = 0 for *i* and *j* from 1 to *L* as the initial conditions. The penalty for indel (*del* = 25.0) was chosen as previously described [46]. The inverse transition matrix was filled together with matrix *F*, in which we determined the maximum element *mF* and built the maximum subsequence from *mF* to the first element of the matrix *F* equal to zero. This maximum subsequence, denoted as *Local*, represented the local alignment of the sequences *S*_1_(*n*) and *S*(*l*), including its coordinates in each sequence.

3. An Iterative Procedure for Finding *F_max_*(*n*)

To determine the best *Local*, we iteratively changed PWM *MT*(*n*, 25). First, we calculated the frequency matrix *M*_1_(*n*, 25) by scanning *Local* from the beginning to end and filling the matrix *M*_1_(*n*, 25) as: *M*_1_(*s*_1_(*i*)*, k*) *= M*_1_(*s*_1_(*i*)*, k*) +1, where *i* is the entire length of *Local*, *s*_1_*(i*) is the *i*-th element of the sequence *S*_1_ in *Local*, and *k* = *s*(*i −* 1) + 5(*s*(*i*) − 1). The sum of the elements in the matrix *M*_1_(*n*, 25) was equal to the length of *Local* minus 1. If, in *Local*, the sequences *S*_1_(*n*) and *S*(*l*) had a missing character, then *i* = *i* + 1 without filling the matrix *M*_1_(*n*, 25). Next, we calculated a new PWM *M*(*n*, 25) using Equation (3), transformed the matrix *M*(*n*, 25), and obtained *MT*(*n*, 25) as described above. *MT*(*n*, 25) was used again in Equation (6) to obtain new *Local* and *mF* values; the procedure was reiterated starting with the construction of a new frequency matrix *M*_1_(*n*, 25) with the new *Local* until the *mF* value was increased. If *mF* remained the same or decreased, or showed cyclical fluctuations, then the iterative procedure was stopped. The maximum value of *mF* corresponding to its *Local* and PWM *MT*(*n*, 25) was obtained as the output.

The above steps were repeated for all matrices from the set *Q*(*n*), for each of which its own *mF*, *Local*, and *MT*(*n*, 25) were created; then the maximum *mF*, denoted as *F_max_(n*), was chosen and used to select *M_max_*(*n*, 25) and *Local*.

#### 4.2.3. Estimation of Statistical Significance for TRs with a Consensus Length of n_0_ by the Monte Carlo Method

To estimate the statistical significance of each TR with a consensus length of *n* using Monte Carlo simulation, we randomly shuffled the sequence *Seq*(*l*) 200 times and obtained a set (*Sr*) containing 200 random sequences, each with a length of *L*. We then took the matrix *M_max_*(*n,* 25), aligned the sequence *S*_1_(*n*) with each sequence from the set *Sr* using the iterative procedure described in Section 4.2.2, and obtained *mF* values, which were inserted into the set *V_n_*. Next, we determined the mean ($\bar{V_{n}})\;$and variance (*D*(*V_n_*)) for the set *V_n_* and calculated the statistical significance *Z*(*n*) of TRs with a consensus length of *n*:

(7) $$Z\left(n\right)=(F_{\max}(n)-\bar{V_{n}})/\sqrt{D(V_{n})}$$

For all consensus lengths, we introduced the same threshold level *Z*_0_, which means that if *Z*(*n*) *> Z*_0_, then the identified TRs can be considered statistically significant.

### 4.3. Filtering of the Obtained Results

For sequence *Seq*(*l*), we obtained a spectrum of *Z*(*n*), and for each *n*, we had *F_max_*(*n*), *M_max_*(*n*, 25), and *Local*. The local alignment coordinates for various values of *n* may differ. If TRs with different lengths *n* overlapped slightly, the protein was considered to have regions with separate TRs, but if the overlap was significant, then the TRs were treated as the same. This situation can be encountered for multiple lengths *n*: 2*n*, 3*n*, etc., or close lengths. In order to avoid duplication of TRs in further analysis, the detected TRs were filtered.

First, for each sequence *Seq*(*l*), we chose the *n_0_* that had *Local_0_* and the largest *Z*(*n*_0_); next, the values of *n* which had a local alignment intersection with *Local_0_* over 50% of the length were excluded, and the next *n_0_* was chosen. The procedure was reiterated until all values of *n* for sequence *Seq*(*l*) had been considered.

### 4.4. Algorithm for TR Classification

The results shown in Table 5 indicated that TRs with consensus lengths of 2 and 7 amino acids were the most common; the former are likely to be associated with β-sheets, as 2 is close to 2.3 residues, corresponding to a typical period for β-structures [4], whereas the latter are known to be located on *α*-helices [36], in which each turn consists of 3.6 residues [48].

We classified all TRs with *Z*(*n*) *> Z*_0_ for *n* equal to 2 and 7 by assessing the difference between the matrices *M_max_*(*n*, 25) for TRs with the same *n*.

The matrix *M_max_*(*n*, 25) can be considered as a point in the *n* × 25 space and the difference between two matrices could be measured according to the Euclidean distance between them according to the formula:

(8) $$B=\sqrt{\sum_{i}^{n}\sum_{j}^{25}\left(m_{\max}^{1}(i,j)-m_{\max}^{2}(i,j\right){)}^{2}}$$

where $m_{\max}^{1}(i,j)$ and $m_{\max}^{2}(i,j)$ are the elements of the matrices *M*^1^_max_(*n*, 25) and *M*^2^_max_(*n*, 25), respectively. It is impossible to know the phase orientation of these matrices; since a phase shift may be present, all distances obtained for any possible phase shifts should be checked. Algorithmically, the phase shift is produced by a cyclic shift of the rows of the second matrix. We take the minimum of *n* calculated distances (*B_min_*) as the resulting distance between two TRs.

For TR consensus lengths *n* = 2 and *n* = 7, we compared all pairs of matrices *M_max_*(2, 25) and *M_max_*(7, 25) and found 2104 and 1650 TRs, respectively; next, the distance matrices *B*_2_(2104, 2104) and *B*_7_(1650, 1650) were calculated. Next, we performed a cluster analysis of the TRs for *n* = 2 and *n* = 7 using the R software environment for statistical computing, with *B*_2_ and *B*_7_ as the input data. First, all TRs were considered to be separate objects and were then merged iteratively into classes with other objects or previous iteration classes according to the shortest distance between them using the complete linkage method. Finally, the algorithm combined all objects into 1 class, forming a hierarchical tree structure.

## Figures and Tables

**Table 1:** Number of TRs with different degrees of evolutionary divergence identified in 5400 artificial sequences by MSHDTR, T-REKS, and XSTREAM.

Divergence Degree *S*, %	Number (Percent) of Detected Sequences with TRs		
	MSHDTR	T-REKS	XSTREAM
25	3194 (59.15)	2997 (55.50)	2622 (48.56)
50	2462 (45.59)	160 (2.96)	39 (0.72)
75	1730 (32.04)	7 (0.13)	4 (0.07)
90	1276 (23.63)	1 (0)	0 (0)
100	949 (17.57)	0 (0)	0 (0)
110	604 (11.19)	0 (0)	0 (0)
120	324 (6.00)	0 (0)	0 (0)
150	42 (0.78)	0 (0)	0 (0)

**Table 2:** Number of TRs detected by MSHDTR, T-REKS, and XSTREAM before and after filtering.

Method	Without Filter	*S* > 0.1	*S* > 0.2	*S* > 0.3	*S* > 0.4	*S* > 0.5
MSHDTR	15,407	15,323	15,223	15,042	14,684	13,986
T-REKS	41,375	37,769	17,599	92	0	0
XSTREAM	19,255	17,305	10,440	5549	871	1

**Table 3:** TRs detected in eight artificial sequences by MSHDTR and HHRepID.

Sequence	Divergence *S*, %	TR Boundaries		MSHDTR				HHRepID			
		Left (*l*_0_)	Right (*r*_0_)	Length, *n*	*l*	*r*	Z	Period	*l*	*r*	e-Value
*SEQ0*	25	161	864	7	161	865	58.4	7	164	866	1.6 × 10^−172^
*SEQ1*	50	14	715	7	161	775	48.8	7	13 380	333 604	5.7 × 10^−54^ 1.9 × 10^−11^
*SEQ2*	75	263	960	7	222	984	22.3	56	373	600	2.1 × 10^−12^
*SEQ3*	90	483	1182	7	445	1227	20.1	7	1103	1144	3.3 × 10^−6^
*SEQ4*	100	254	959	7	220	1244	17.7	15 14	20 1062	48 1086	2.6 × 10^−5^ 2.7 × 10^−5^
*SEQ5*	110	553	1254	7	385	1254	13.5	-			
*SEQ6*	120	144	839	7	130	848	11.3	-			
*SEQ7*	150	214	915	7	1	1188	5.0	-			

**Table 4:** The 10 most common consensus lengths of TRs detected in amino acid sequences from Swiss-Prot.

Consensus Length, *n*	Number of Detected TRs	% of TRs
2	2104	13.65
3	1349	8.75
4	1205	7.82
5	1180	7.66
6	1005	6.52
7	1650	10.71
8	759	4.92
9	699	4.54
10	450	2.92
11	528	3.43

**Table 5:** Multiple alignment of 64-character repeats found in the sequence of *Mus musculus* hexokinase-1 (P17710).

No.	Sequence of a Period
1	NMIMMTN..MNK.KN.MTIN…KNK.NKMNKKNIKMNKKTKNTMKTI.KKKTK.KKNN.IMKKMKNK.NMTM.NNNKNNT
2	NTKITNK..TNK.KM.KNKN…N.K..ITMKKTN.KKTINTMMMKMT.MMKKKKININ.IKN.M.N..NMKT.TKKKKNI
3	NMMIMKNKKTKK.TKKMKKN…MKK.MMMKTITK.NKKKKKNTNKKN.NNNNK.ITTN.NNT.K.K..KKKK.NKNNKNI
4	NTTKMMK..TKK.TK.TTKM…NNK.NNTIKKIK.TTKNKTTKMNTI.TMTKT.MKNK.NKK.M.N..KITM.NKNK.NI
5	NKTKKMK..K.K.KM..NKM…TNK.KITKMKNK.TKKNMKMINNNT.KKKKTNTMTKKNNK.M.TKKNMKKKTKNMTTN
6	KNKNMKN..NKK.NI.MNKN…KKKKNKKKKKNM.KMTNMKNKMKMN.NKKKT….K.NKI.M.N..MKNI.NMMIMMN
7	KMMKKK…TNT.KM.IKKN…TNK.NKMKKKN….KNKKKIMKKT.NMMNK.TTNK.NMI.M.K..NM…NKKNTKM
8	MMKMNTN..TNK.KM.MTNN…NMK.NKMNKKNIKMNKKTKNTMKTI.KKKTK.KKNN.IMK.K.K..KMKM.NKMMMNK
9	TNMNMKI..NKKKTK.NNKN…KTT.KITMKKNN.KNTIKTINKKMKKMNKKK.ININ.IKN.M.N..NNKT.NKKKKNI
10	NMKIMKN..TNK.KM.TKKNKKMTKK.MMMTTITK.TKKKKKNTNKKN.NNNNK.ITTK.NNT.K.K..KKKK.NKNNKNI
11	NTTNMNK..TNK.TK.NNKN…NNK.NNTIKKIK.TNKNKTTKMNTI.TMKKT.TINK.NNK.M.N..MITM.NKNK.NI

**Table 6:** Multiple alignment of six-character repeats found in aspartyl/glutamyl-tRNA (Asn/Gln) amidotransferase subunit B (Q02GV7) from Pseudomonas aeruginosa.

No.	Repeat Sequence	No.	Repeat Sequence
1	N.K.T..K.N.K.	25	M.K.N….K.K.
2	M.N.T..N.N.K.	26	N.K.T..K.M.K.
3	N.K.M..K.M.K.	27	K.K.N..I.K…
4	N.K.T..I.K.N.	28	N.K.T..KKN.KK
5	M.K.T..K.M…	29	N.M.T..K.K.T.
6	N.K.N..N.I.M.	30	K.T.N..N.K.K.
7	I.K.T..M.K.K.	31	N.K.TN.K.K.K.
8	N.M.T..K.N.M.	32	N.K……K.M.
9	N.K.T..K.K.T.	33	K.M.T..N.N.K.
10	T.K.K..M.K.K.	34	N.K.M..K.K.M.
11	N.T.N..M.K.I.	35	N.KIT..K.N.K.
12	T.K.N..M.T.T.	36	N.K.T..K.NKT.
13	N.M.N..N.M.K.	37	N.KKT..K.M.K.
14	M.T.T..K.N.T.	38	K.M.N..K.N.T.
15	I.M.I..I.K…	39	N.K.K..K.T.M.
16	N.K.T..K.K.K.	40	N.K.TTKK.K.K.
17	K.KKT..K.T.I.	41	N.K.T..N.K.T.
18	K.K.M..K.M.T.	42	N.I.M..K.K.T.
19	N.KKT..K.K.K.	43	T.K.M..M.K.M.
20	N.M.M..T.M.I.	44	NIK.I..I.K.K.
21	T.N.N..I.K.K.	45	N.K.N..M.K.N.
22	N.KIT..K.N.KK	46	M.K.M..K.N.K.
23	N.K.N..M.T…	47	N…………
24	N.K.T..I.I.T.		

## Supplementary Materials

The following are available online at https://www.mdpi.com/article/10.3390/ijms22137096/s1.
