# Supplementary material for: Search for Highly Divergent Tandem Repeats in Amino Acid Sequences
Source: Int J Mol Sci. 2021 Jul 1;22(13):7096. doi: 10.3390/ijms22137096 (PMC8269118; doi:10.3390/ijms22137096)
Supplement: Supplementary file 1 [file ijms-22-07096-s001.zip › ijms-1254136-supplementary.pdf]

SUPPLEMENTED MATERIALS

Matrices of 19 classes obtained as a result of classification of TRs with consensus length 2. Number of elements in each class: {3, 6, 178, 122, 23, 98, 12, 30, 767, 87, 14, 86, 95, 108, 13, 112, 176, 32, 142}. Matrix element (p, $a_i a_j$), a_i,a_j =*{K, N, I, M, T}* should be interpreted as a_i before a_j , a_j in consensus position p. Symbols *{K, N, I, M, T}* were obtained by re-coding of amino acid sequences according to side-chain polarity: K = {G, *A, V, I, L, P}; N = {S, T, C, M, Q, N}; I = {F, Y, W}; M = {K, R, H}; T = {D, E}*

CLASS0

p	KK	KN	KI	KM	KT	NK	NN	NI	NM	NT	IK	IN	II	IM	IT	MK	MN	MI	MM	MT	TK	TN	TI	TM	TT
1	-0,23	0,4	0,27	0,1	-0,9	-0,03	3,03	2,13	-2,27	0,97	-0,27	0,37	2,27	-1,07	-0,4	-6,67	-1,93	-1,17	2,83	-1,67	2,63	0,23	-0,43	-1,07	1,33
2	-2,33	1,6	1,73	-1,77	0,3	0,8	1,63	0,9	-2	0,1	-0,27	2,77	-0,4	-0,93	0,03	-3,4	-1,93	-1,5	1,8	1,13	-0,37	0,37	-0,43	-1,07	-0,47

CLASS1

p	KK	KN	KI	KM	KT	NK	NN	NI	NM	NT	IK	IN	II	IM	IT	MK	MN	MI	MM	MT	TK	TN	TI	TM	TT
1	-1,32	-0,93	1,18	1,3	-0,27	-5,42	2,13	-1,17	0,03	-1,77	2,62	-0,72	-0,1	0,4	-0,43	-0,72	-1,15	-0,18	0,13	0,9	1,55	-0,33	0,42	0,62	0,95
2	1,57	-2,82	0,6	0,35	2,17	-5,78	3,27	-0,75	-0,95	-1	0,13	-1,6	0,85	0,1	1,43	0,12	-1,52	2,17	0,02	3,83	-0,85	-1,27	0,53	-0,17	-0,68

CLASS2

p	KK	KN	KI	KM	KT	NK	NN	NI	NM	NT	IK	IN	II	IM	IT	MK	MN	MI	MM	MT	TK	TN	TI	TM	TT
1	-0,27	-3,79	0,06	0,41	0,04	-4,29	3,18	-0,76	-0,99	-1,12	0,36	-0,82	0,31	0,43	0,52	0,15	-1,89	0,21	1,32	0,53	-0,02	-1,42	0,46	0,51	1,34
2	-0,36	-0,21	1,06	0,8	1,02	-7,74	2,96	-0,85	-1,51	-1,41	-0,46	-0,74	0,34	0,21	0,27	-0,57	-0,92	0,27	1,09	0,72	-0,7	-1,05	0,61	0,41	0,98

CLASS3

p	KK	KN	KI	KM	KT	NK	NN	NI	NM	NT	IK	IN	II	IM	IT	MK	MN	MI	MM	MT	TK	TN	TI	TM	TT
1	-2,14	0,6	0,87	1,56	0,5	-6,98	2,8	-0,88	-0,99	-1,8	-0,56	-0,74	0,32	0,5	0,54	-1,47	-1,33	0,1	1,21	-0,14	-1,03	-1,52	0,49	0,34	2,21
2	-2,42	-2,77	0,1	-0,16	-0,05	-3,49	2,71	-0,55	-1,28	-1,38	0,14	-0,88	0,32	-0,02	0,51	0,53	-1,11	0,51	1,1	0,46	-0,46	-1,79	0,2	-0,11	2,1

CLASS4

p	KK	KN	KI	KM	KT	NK	NN	NI	NM	NT	IK	IN	II	IM	IT	MK	MN	MI	MM	MT	TK	TN	TI	TM	TT
1	-2,09	-0,57	0,24	-0,47	0,59	-4,88	2,46	-0,39	-1,3	-1,01	0,22	-0,3	0,24	-0,1	0	-1,21	-1,44	0,17	0,39	4,14	-1,7	-1,69	-0,13	-0,66	0,09
2	-2,5	-0,1	0,01	-0,31	-0,26	-4,72	2,32	-0,21	-1,01	-1,89	-0,05	-0,38	-0,3	0,18	-0,29	-1,33	-1,45	-0,17	0,07	-0,51	-1,23	-1,51	1,45	4,22	0,45

CLASS5

p	KK	KN	KI	KM	KT	NK	NN	NI	NM	NT	IK	IN	II	IM	IT	MK	MN	MI	MM	MT	TK	TN	TI	TM	TT
1	-5,32	-0,99	-0,45	-2,24	-0,88	-1,81	0,52	-0,14	-1,04	-0,2	0,37	0,2	0,02	-0,48	-0,19	3,94	1	0,01	-1,21	0,12	0,13	-0,25	0,07	-0,92	0,41
2	-5,23	0,18	0,72	5,77	1,4	-3,19	0,66	-0,01	0,8	0,12	-0,85	0,09	-0,15	-0,04	-0,11	-3,86	-1,09	-0,24	-1,26	-1	-2,26	-0,13	0,19	0,34	0,11

CLASS6

p	KK	KN	KI	KM	KT	NK	NN	NI	NM	NT	IK	IN	II	IM	IT	MK	MN	MI	MM	MT	TK	TN	TI	TM	TT
1	-5,44	2,29	6	1,99	0,47	-3,54	-0,08	-0,02	0,83	0,22	-3,24	-0,86	-1,09	-0,97	-0,66	-1,97	-0,48	-0,77	0,49	-0,1	-1,14	0,14	0,13	-0,26	0,32
2	-5,71	-1,44	-1,85	-0,99	-0,65	0,47	-0,08	-1,22	-0,51	0,62	3,99	-0,14	-1,32	-0,72	0,24	1,47	-0,01	-0,65	-0,1	-0,02	-0,48	0,34	0	0,16	-0,46

CLASS7

p	KK	KN	KI	KM	KT	NK	NN	NI	NM	NT	IK	IN	II	IM	IT	MK	MN	MI	MM	MT	TK	TN	TI	TM	TT
1	-3,69	-2,39	-0,3	-0,88	-0,9	-2,26	1,64	0,2	-0,86	-0,13	0,17	-0,52	0,11	-0,01	-0,27	3,49	-0,96	0,04	-0,09	-0,37	-1,05	-0,94	0,03	-1	1,38
2	-4,24	4,23	0,03	0,69	0,67	-5,39	-0,82	0,16	1,42	-0,4	-0,84	0,42	0,03	-0,2	-0,05	-2,33	-0,81	-0,18	-0,48	-0,71	-2,35	-0,36	-0,07	0,82	0,54

CLASS8

p	KK	KN	KI	KM	KT	NK	NN	NI	NM	NT	IK	IN	II	IM	IT	MK	MN	MI	MM	MT	TK	TN	TI	TM	TT
1	-5,72	6,18	0,6	1,35	1,27	-5,54	-1,18	-0,57	-0,76	-0,82	-1,32	0,44	-0,08	0,13	0,04	-1,95	0,09	0,12	-0,08	0,05	-1,78	0,05	0,14	0,13	0,01
2	-5,73	-2,76	-0,66	-0,81	-0,79	3,18	-1,17	0,49	0,13	0,19	0,03	-0,64	-0,08	-0,1	0,04	0,15	-0,72	0,12	-0,09	0,02	0,25	-0,95	0,06	0,1	0,01

CLASS9

p	KK	KN	KI	KM	KT	NK	NN	NI	NM	NT	IK	IN	II	IM	IT	MK	MN	MI	MM	MT	TK	TN	TI	TM	TT
1	-5,42	-1,04	-0,24	-0,96	-2,21	-1,41	0,04	0,31	0,55	-1,02	-0,57	0,17	0,06	-0,13	-0,31	-0,32	-0,01	0,22	0,24	-0,87	4,7	0,43	-0,04	-0,21	-1,4
2	-5,54	1,3	0,26	1,08	5,94	-3,31	0,27	-0,11	0,04	0,58	-0,82	0,3	0,08	0,1	0,2	-1,86	-0,05	-0,07	0,24	-0,08	-3,81	-1,12	-0,33	-0,99	-1,3

CLASS10

p	KK	KN	KI	KM	KT	NK	NN	NI	NM	NT	IK	IN	II	IM	IT	MK	MN	MI	MM	MT	TK	TN	TI	TM	TT
1	-4,96	4,29	-0,06	0,19	1,1	-4,09	-0,62	-0,58	-0,74	0,01	-1	0,04	-0,21	1,37	-0,13	-2,44	-0,44	-0,19	-0,6	2,7	-1,71	-0,84	-0,27	-0,21	-0,81
2	-4,53	-1,61	-0,4	-0,61	-0,43	-3,14	0,08	2,79	1,17	-0,83	-1,34	-0,58	-0,57	0,51	-0,31	0,11	-0,84	-0,24	-0,07	-0,74	3,43	-0,55	-0,18	-0,4	-0,81

CLASS11

p	KK	KN	KI	KM	KT	NK	NN	NI	NM	NT	IK	IN	II	IM	IT	MK	MN	MI	MM	MT	TK	TN	TI	TM	TT
1	-3,53	1,6	0,01	1,51	-1,46	-3,38	0,76	0,05	0,04	-1,7	-0,32	0,22	0,33	0,12	-0,41	-1,64	0,79	-0,02	0,96	-1,57	-4,27	-1,12	-0,34	-1,56	3,71
2	-3,93	0,42	-0,04	0,06	-1,59	-0,98	0,33	0,46	0,32	-0,92	-0,45	-0,13	0,1	0,14	-0,47	-0,73	0,09	0,14	1,49	-1,58	-3,94	-1,93	-0,1	-1,53	3,55

CLASS12

p	KK	KN	KI	KM	KT	NK	NN	NI	NM	NT	IK	IN	II	IM	IT	MK	MN	MI	MM	MT	TK	TN	TI	TM	TT
1	-3,76	2,09	0,35	0,48	0,06	-4,73	-1,5	-0,56	-2,4	-0,94	-0,59	0,55	-0,13	0	0,24	-3,78	5,4	-0,37	-0,85	0,01	-1,85	0,35	-0,04	-0,29	0,56
2	-4,42	-1,57	-0,27	-0,52	-0,21	1,18	-1,53	0,78	3,5	0,51	-0,89	-0,41	0,07	-0,31	0,02	-3,51	-2,4	-0,02	0,22	-0,54	-1,67	-1,07	0,3	0,2	0,59

CLASS13

p	KK	KN	KI	KM	KT	NK	NN	NI	NM	NT	IK	IN	II	IM	IT	MK	MN	MI	MM	MT	TK	TN	TI	TM	TT
1	-4,16	0,1	-0,02	0,37	-0,77	-2,6	0,48	0,22	0,5	-0,98	-0,48	0,12	0,09	0,11	-0,25	-2,89	-0,89	-0,4	-1,73	-2,04	-1,7	-0,02	0,12	6,51	-1,83
2	-3,89	0,45	0,49	-0,64	0,37	-3,04	0,57	0,08	-0,86	0,11	-0,83	0,08	0,07	-0,25	0,14	-1,8	0,34	0,17	-1,63	6,31	-2,96	-0,8	-0,4	-2,18	-1,75

CLASS14

p	KK	KN	KI	KM	KT	NK	NN	NI	NM	NT	IK	IN	II	IM	IT	MK	MN	MI	MM	MT	TK	TN	TI	TM	TT
1	-4,53	2,49	0,06	2,42	-0,96	-3,75	-0,27	0,25	-0,07	-1,34	-1,16	-0,2	0,04	-0,31	-0,62	-3,56	-0,51	-0,36	-0,76	-1,52	-2,51	1,27	1,48	2,86	-0,48
2	-4,12	-1,03	0,16	-1,11	-0,92	0,33	0,27	0,15	-0,21	-0,34	0,88	0,14	-0,16	-0,55	0,55	-2,65	0,04	-0,75	-0,77	5,25	-2,77	-1,69	-0,69	-1,66	-0,33

CLASS15

p	KK	KN	KI	KM	KT	NK	NN	NI	NM	NT	IK	IN	II	IM	IT	MK	MN	MI	MM	MT	TK	TN	TI	TM	TT
1	-3,84	0,22	0,51	0,16	-0,68	-2,1	-0,23	0,16	2,39	-0,87	-0,87	-0,21	-0,08	0,08	-0,31	-3,59	-1,45	-0,37	-1,55	-2,21	-1,89	-0,41	0,35	5,65	-1,45
2	-3,87	0,42	-0,16	-0,67	0,54	-2,62	-0,27	-0,05	-1,55	-0,37	-0,3	-0,14	0,18	-0,26	0,63	-2,46	2,97	-0,05	-1,62	5,33	-2,82	-1,04	-0,34	-2,23	-1,31

CLASS16

p	KK	KN	KI	KM	KT	NK	NN	NI	NM	NT	IK	IN	II	IM	IT	MK	MN	MI	MM	MT	TK	TN	TI	TM	TT
1	-3,17	0,52	0,18	-1,07	0,5	-4,34	-1,21	-0,28	-2,65	-0,61	-0,28	0,48	-0,15	-0,44	0,05	-2,2	5,78	-0,04	-1,38	1,41	-1,69	-0,18	-0,01	-1,01	-0,03
2	-3,68	-0,98	0,13	0,96	-0,08	-2,04	-1,25	0,46	5,38	-0,13	-0,59	-0,43	-0,05	0,12	-0,14	-3,88	-2,57	-0,36	-1,54	-0,93	-1,46	-0,58	0,13	1,63	-0,22

CLASS17

p	KK	KN	KI	KM	KT	NK	NN	NI	NM	NT	IK	IN	II	IM	IT	MK	MN	MI	MM	MT	TK	TN	TI	TM	TT
1	-4,41	-0,24	-0,11	-0,41	-1,06	-4,11	-0,02	-0,76	-1,18	-1,61	0,38	0,37	-0,07	-0,55	-0,13	-0,68	0,51	0,13	0,42	-1,24	0,8	3,46	-0,27	0,18	-0,89
2	-4,24	0,21	-0,02	1,07	2,18	-3,79	-0,3	1,42	2,96	1,11	-1,18	-0,89	-0,27	-0,42	0,63	-2,37	-0,82	-0,17	-1,03	1,4	-2,49	-2,04	-0,32	-1,38	-0,34

CLASS18

p	KK	KN	KI	KM	KT	NK	NN	NI	NM	NT	IK	IN	II	IM	IT	MK	MN	MI	MM	MT	TK	TN	TI	TM	TT
1	-3,49	0,95	0,07	0,2	-0,89	-4,65	-1,54	-0,21	-0,44	-2,45	-0,56	0,09	-0,05	-0,04	-0,2	-1,3	0,04	0,09	0,29	-0,71	-1,56	5,88	0,13	0,31	-1,48
2	-3,33	-0,89	0,02	0,17	0,52	-3,24	-1,51	0,1	-0,04	6,42	-0,41	-0,25	-0,01	-0,09	0,22	-0,94	-0,41	-0,01	0,45	-0,2	-3,04	-2,46	-0,2	-0,67	-1,61

1. Matrices of 8 classes obtained as a result of classification of TRs with consensus length 7. Number of elements in each class: {45, 49, 146, 393, 66, 514, 158, 279}. Matrix element (p, $a_i a_j$), $a_i, a_j = \{K,N,I,M,T\}$ should be interpreted as a_i before a_j , a_j in consensus position p. Symbols *{K, N, I, M, T}* were obtained by re-coding of amino acid sequences according to side-chain polarity: *K = {G, A, V, I, L, P}; N = {S, T, C, M, Q, N}; I = {F, Y, W}; M = {K, R, H}; T = {D, E}*

CLASS0

p	KK	KN	KI	KM	KT	NK	NN	NI	NM	NT	IK	IN	II	IM	IT	MK	MN	MI	MM	MT	TK	TN	TI	TM	TT
1	-3,89	-0,52	-0,16	-1,06	0,66	-1,81	-0,22	0,49	0,16	0,39	-0,33	0,49	-0,1	-0,14	0,05	-0,35	-0,28	0,03	-0,6	0,53	-2,38	-0,5	-0,09	-0,96	0,12
2	-4,89	-0,38	0,4	0,09	3,56	-3,51	-0,35	0,24	-0,75	0,67	-0,25	-0,23	-0,16	-0,06	0,24	-2,62	-0,49	-0,28	-0,81	-0,46	-1,47	-0,6	-0,2	-0,55	2,57
3	-4,6	-0,32	0,22	-0,75	-0,44	-2,98	-0,51	0	-0,62	-0,8	-0,14	-0,25	-0,01	-0,08	-0,14	-1,85	-0,26	-0,23	-1,02	-1	1,56	3,11	0,02	0,05	-0,02
4	-4,21	-0,32	-0,16	2,78	0,03	-3,12	1,48	-0,08	0,48	-0,06	-0,21	0,06	-0,07	-0,36	-0,01	-2,83	-0,57	-0,19	-0,15	-0,57	-2,78	-0,71	0,11	-0,27	-0,49
5	-4,78	-0,37	-0,14	-0,58	-0,45	-3,3	0,8	0,04	-0,05	0,02	-0,67	-0,16	-0,02	0,11	-0,07	-2,2	0,1	0,02	4,18	-0,58	-2,35	-0,69	-0,13	0,09	0,25
6	-4,04	-0,58	-0,24	-0,56	-0,99	-2,06	-0,25	-0,17	-0,53	-0,08	-0,28	0,09	-0,03	-0,36	-0,13	0,66	0,82	0,46	0,61	-0,63	-1,74	0,56	-0,05	-0,68	-0,65
7	-3,61	1,16	0,27	1,75	0,08	-3,03	0,57	0,3	0,27	-0,14	-0,38	0	-0,1	0,14	-0,13	-2,52	0,08	0,06	0,15	-0,94	-2,02	-0,42	-0,06	-1,01	-0,67

CLASS1

p	KK	KN	KI	KM	KT	NK	NN	NI	NM	NT	IK	IN	II	IM	IT	MK	MN	MI	MM	MT	TK	TN	TI	TM	TT
1	-3,37	0,97	1,47	3,88	-0,44	-2,84	-0,33	0,92	1,21	-0,48	-0,88	-0,02	-0,28	0,13	-0,37	-2,93	-0,58	-0,42	0,44	-0,74	-2,97	-0,89	-0,21	-0,56	-1,1
2	-4,32	-0,53	0,3	-0,22	-0,51	-3,11	-0,11	-0,29	-0,15	-0,03	-1,06	0,24	0,38	0,75	0,39	-2,53	0,87	-0,16	0,12	4,42	-3	-0,81	-0,12	-0,5	-0,83
3	-4,69	-0,29	-0,19	-0,7	-0,54	-2,77	0,25	-0,25	-0,51	0,03	-0,88	-0,07	-0,18	0,19	0,15	-2,6	0,37	-0,27	-0,3	0,08	-1,23	2,17	0,18	-0,4	0,21
4	-4,18	0,02	0,65	-0,68	0	-2,66	0,14	0,6	-0,56	2,46	-0,78	-0,19	-0,12	-0,4	-0,11	-3,02	-0,54	-0,26	-0,82	0,33	-1,54	-0,19	0,38	-0,71	0,17
5	-4,09	-0,27	-0,08	-0,35	0,66	-2,44	-0,43	-0,25	-0,38	0,11	0,08	0,49	-0,16	-0,08	0,1	-2,93	-0,57	-0,21	-0,87	-0,8	-2,19	-0,43	-0,23	-0,18	3,03
6	-4,14	0,36	-0,26	-0,12	0,01	-3,2	0,06	0,06	-0,36	-0,47	-0,91	-0,17	-0,06	-0,28	-0,26	-2,88	-0,58	-0,31	0,08	-0,7	-2,57	0,43	-0,11	4,3	-0,83
7	-3,91	-0,33	-0,25	-0,73	-0,87	-1,85	0,47	0,04	-0,3	-0,86	-0,89	-0,17	-0,13	-0,2	-0,15	2,37	1,2	0,06	-0,15	-0,81	-2,12	-0,72	-0,32	-0,55	-0,9

CLASS2

p	KK	KN	KI	KM	KT	NK	NN	NI	NM	NT	IK	IN	II	IM	IT	MK	MN	MI	MM	MT	TK	TN	TI	TM	TT
1	-4,66	0,22	0,55	0,69	1,29	-2,65	-0,21	0,18	0,05	-0,23	-1,03	-0,18	-0,06	-0,11	-0,09	-1,85	-0,28	0,08	-0,1	0,07	-1,08	-0,25	-0,04	0,13	-0,13
2	-5,15	-0,61	0,24	-0,05	0,19	-2,57	-0,26	0,12	-0,3	0,29	-0,38	-0,19	0,07	0,2	0,73	-0,61	-0,04	0,35	-0,23	0,18	-0,24	-0,1	0,17	0,15	0,05
3	-3,26	-0,9	0,26	-0,66	0,2	-2,26	-0,61	-0,13	-0,55	-0,27	0,17	-0,13	-0,14	-0,13	0,11	-0,84	-0,59	-0,07	-0,39	-0,2	0	-0,33	0,26	-0,1	-0,14
4	-5,85	5,71	-0,11	-0,33	0,06	-3,85	0,45	-0,44	-0,59	-0,48	-1,17	1,24	-0,14	-0,24	-0,12	-2,39	0,26	-0,34	-0,3	-0,42	-1,95	0,94	-0,17	-0,21	-0,01
5	-5,61	-1,63	-0,6	-1,19	-1,02	5,99	-0,5	0,12	-0,21	0,2	-0,53	-0,54	-0,27	-0,57	-0,26	-1,46	-0,6	-0,25	-0,53	-0,39	-0,75	-0,68	-0,35	-0,45	-0,36
6	-4,9	1,46	1,33	5,57	1,39	-3,98	-0,99	-0,33	-0,06	-0,57	-1,4	-0,37	0,08	0,11	-0,13	-2,53	-0,78	-0,13	-0,27	-0,37	-2,02	-0,62	-0,05	-0,02	-0,27
7	-5,75	-1,23	-0,61	-0,93	-0,63	-2,73	-0,06	-0,19	-0,13	0,03	0,11	0,17	0	0,01	-0,03	2,36	0,97	0,18	0,54	0,6	-0,74	-0,05	0,1	-0,11	0,15

CLASS3

p	KK	KN	KI	KM	KT	NK	NN	NI	NM	NT	IK	IN	II	IM	IT	MK	MN	MI	MM	MT	TK	TN	TI	TM	TT
1	-5,32	-0,84	-0,29	-0,29	0,21	-4,07	0,49	0,06	0,85	1,59	-1,11	0	-0,02	-0,02	0,05	-2,59	0,06	-0,03	0,57	1,03	-2,62	0,29	-0,12	0,53	0,91
2	-3,94	-1,77	-0,18	-1,05	-1,39	-0,65	-1,15	0,33	-0,81	-1,17	-0,21	-0,51	0,01	-0,22	-0,27	1,02	-0,65	0,58	-0,48	-0,82	2,75	-0,46	0,28	-0,16	-0,84
3	-3,37	5,08	0,3	1,86	2,15	-4,76	-1,03	-0,4	-0,64	-0,86	-0,79	0,55	-0,06	0,27	0,33	-2,96	-0,52	-0,34	-0,5	-0,36	-3,19	-1,09	-0,43	-0,86	-0,89
4	-5,32	-0,9	-0,39	-0,49	-0,29	-3,83	1,34	0,21	1,07	1,78	-1,1	-0,24	-0,11	-0,03	-0,05	-2,53	0,12	-0,1	0,27	0,71	-2,66	0,21	0,02	0,46	0,74
5	-4,1	-1,55	0,03	-1,03	-1,25	-0,78	-0,73	0,46	-0,61	-0,95	-0,37	-0,26	-0,04	-0,12	-0,36	0,27	-0,41	0,48	-0,4	-0,59	0,99	0,01	0,37	-0,04	-0,47
6	-4,04	3,31	0,3	2,36	1,55	-4,43	-0,55	-0,17	-0,37	-0,62	-0,75	0,61	0,02	0,43	0,4	-2,54	-0,66	-0,1	-0,54	-0,27	-2,94	-0,91	-0,32	-0,57	-0,8
7	-5,16	-0,79	-0,27	-0,55	-0,44	-3,64	1,3	0,14	0,64	0,52	-0,94	0,14	-0,06	0	-0,02	-2,19	0,83	-0,01	0,35	0,63	-2,18	0,3	-0,18	0,31	0,1

CLASS4

p	KK	KN	KI	KM	KT	NK	NN	NI	NM	NT	IK	IN	II	IM	IT	MK	MN	MI	MM	MT	TK	TN	TI	TM	TT
1	0,72	0,54	0,67	0,87	1,19	-4,21	-1,32	-0,5	-0,95	-0,57	-1,01	-0,41	-0,12	-0,41	-0,09	-2,37	-0,88	-0,27	-0,37	-0,19	-1,96	-0,78	-0,35	-0,45	-0,65
2	-5,21	1,63	0,41	1,15	1,27	-4,12	-0,45	-0,25	-0,16	-0,12	-0,98	-0,06	0,22	-0,25	0,18	-2,27	-0,23	0,07	0,12	-0,01	-1,87	-0,09	0,15	-0,06	0,73
3	-5,55	-1,23	-0,14	-0,66	-0,37	-2,97	-0,21	0,74	0,47	0,99	-0,52	0,08	-0,07	0,21	0,08	-1,04	-0,27	0,34	0,28	0,5	-0,42	-0,14	0,38	0,73	0,4
4	-4,95	-0,21	0,07	0,21	0,57	-3,57	-0,75	0	-0,32	-0,02	-0,31	0,15	0,26	0,06	0,34	-0,97	-0,08	0,49	0,15	-0,09	-0,02	-0,27	0,64	-0,09	0,02
5	-5,2	0,41	-0,16	0,77	0,08	-3,52	-0,32	-0,36	0,35	-0,25	-0,18	0	-0,02	1	0,07	-1,99	-0,25	0	0,49	0,26	-1,56	0,03	0,1	1,39	0,01
6	-6,02	1,22	-0,13	-0,66	-0,75	-4,42	2,48	-0,45	-0,63	-0,24	-1,08	0,36	-0,26	-0,21	-0,28	-2,38	4,7	0,04	-0,22	-0,07	-2,07	1,34	-0,19	-0,14	-0,33
7	-5,58	-1,66	-0,58	-1,08	-1,07	6,7	-0,75	-0,06	-0,19	-0,45	-0,68	-0,62	-0,17	-0,26	-0,35	-1,58	-1,07	-0,25	-0,43	-0,4	-1,02	-0,82	-0,18	-0,61	-0,65

CLASS5

p	KK	KN	KI	KM	KT	NK	NN	NI	NM	NT	IK	IN	II	IM	IT	MK	MN	MI	MM	MT	TK	TN	TI	TM	TT
1	-3,82	4,68	0,26	1,45	4,16	-4,59	-0,98	-0,34	-0,86	-0,42	-0,98	0,36	0,02	0,48	0,43	-2,92	-0,82	-0,34	-0,82	-0,69	-3,43	-1,01	-0,44	-0,8	-0,91
2	-5,7	-0,81	-0,34	-0,48	-0,98	-3,05	1,27	0,15	1,09	0,62	-0,89	-0,05	-0,11	-0,12	-0,17	-2,43	0,02	-0,05	0,34	-0,05	-1,91	0,86	0,1	1,07	0,99
3	-5,34	-0,61	-0,21	-0,23	-0,51	-3,23	0,74	0,08	0,96	0,16	-0,69	0,11	-0,04	-0,19	-0,08	-1,48	0,74	0,18	0,75	0,09	-2,41	0,13	0,02	0,7	0,18
4	-3,99	-1,02	-0,03	-0,08	-1,24	-0,57	-0,81	0,24	0,03	-0,97	-0,4	-0,27	0,14	-0,04	0,03	0,9	-0,34	0,88	-0,34	-0,63	-0,53	-0,77	0,18	-0,09	-1,04
5	-4,33	1,96	0,15	1,72	3,06	-4,1	-0,77	-0,36	-0,4	-0,37	-0,81	0,32	-0,03	0,37	0,85	-2,29	0,15	-0,18	0,23	-0,19	-3,3	-0,9	-0,3	-0,76	-0,74
6	-5,64	-0,57	-0,41	-0,65	-0,44	-3,67	0,97	-0,03	0,02	0,72	-0,86	-0,07	-0,11	-0,15	-0,14	-2,11	0,6	-0,04	0,1	0,88	-2,46	0,91	0,21	0,52	1,86
7	-3,52	-1,58	-0,27	-1,28	-1,47	1,07	-0,91	0,29	-0,89	-0,99	0,04	-0,51	-0,04	-0,27	-0,41	-0,03	-0,83	0,43	-0,66	-0,92	2,2	-0,62	0,4	-0,66	-0,82

CLASS6

p	KK	KN	KI	KM	KT	NK	NN	NI	NM	NT	IK	IN	II	IM	IT	MK	MN	MI	MM	MT	TK	TN	TI	TM	TT
1	-5,14	6,09	0,28	0,26	0,93	-4,2	-0,24	-0,48	-0,71	-0,6	-1,27	0,12	-0,2	-0,25	-0,11	-2,43	0	-0,2	-0,44	-0,29	-2,25	0,32	-0,21	-0,31	-0,37
2	-5,56	-1,78	-0,42	-0,91	-0,96	3,33	0,27	0,41	-0,13	-0,01	-0,71	-0,19	-0,12	-0,3	-0,11	-1,28	-0,61	-0,2	-0,53	-0,36	-0,77	-0,46	-0,15	-0,13	-0,32
3	-2,86	1,46	0,6	0,81	1,15	-3,56	-0,59	-0,04	-0,52	-0,55	-0,52	-0,22	-0,08	-0,24	-0,12	-1,93	-0,58	-0,08	-0,32	-0,35	-1,81	-0,7	0	-0,35	-0,27
4	-5,36	0,04	0,07	0,26	1,06	-3,42	0,94	-0,01	0,14	0,17	-0,66	0,37	0,16	0,16	0,07	-1,38	0,04	0,09	-0,07	-0,08	-1,5	0,35	0,07	0,21	0,16
5	-5,03	-0,85	-0,38	-0,46	-0,37	-1,18	0,01	0,07	-0,15	-0,02	-0,42	0,16	-0,12	0	-0,2	-0,71	0,01	-0,01	-0,07	-0,08	-0,26	0,14	0,05	0,18	0,13
6	-5,37	-0,49	0,95	3,05	1,11	-3,68	-0,43	0,17	0,86	0,44	-1,06	-0,33	0,18	0,31	0,18	-2,15	-0,22	0,25	0,1	0,22	-2,09	-0,4	0,22	0,7	0,21
7	-5,07	-1,5	-0,55	-0,94	-0,79	-2,15	-0,97	-0,39	-0,65	-0,62	0,81	0,02	-0,13	0,01	0,06	3,5	-0,32	0,1	-0,14	0,12	1,31	-0,57	-0,1	-0,23	-0,13

CLASS7

p	KK	KN	KI	KM	KT	NK	NN	NI	NM	NT	IK	IN	II	IM	IT	MK	MN	MI	MM	MT	TK	TN	TI	TM	TT
1	-4,01	0,97	0,6	1,31	3	-4,04	-0,95	-0,31	-0,44	-0,25	-1,2	-0,18	-0,15	0,06	0,24	-1,86	-0,29	-0,03	-0,11	0,03	-1,74	-0,43	0	-0,05	-0,04
2	-5,25	-0,83	-0,05	-0,19	-0,4	-2,85	-0,21	0,13	-0,03	-0,11	-0,63	0,01	0,09	0,03	-0,04	-0,53	0,14	0,29	0,01	-0,08	1,03	0,07	0,34	0,44	0,15
3	-4,32	0,21	0,37	0,51	0,37	-3,12	-0,44	0,18	-0,08	0,06	-0,48	0,13	0,05	0,09	0,18	-1,26	-0,04	0,16	0,02	0,14	-1,52	-0,21	0,11	0,04	-0,15
4	-5,25	-0,48	-0,05	0,45	0,72	-3,26	0,21	0,07	0,34	0	-0,6	0,28	0,04	0,44	0,07	-1,37	0,38	0,19	0,34	0,06	-1,06	0,12	0,1	0,52	-0,15
5	-3,76	-1,48	-0,27	-0,46	-0,67	-0,83	-1,01	-0,3	-0,44	-0,48	0,17	-0,52	-0,27	-0,15	-0,13	0,82	-0,52	-0,02	-0,07	-0,09	0,48	-0,66	-0,19	-0,17	-0,3
6	-5,73	7,73	0,08	0,05	-0,09	-4,43	-0,43	-0,36	-0,73	-0,71	-1,44	0,28	-0,05	-0,15	-0,25	-2,15	0,32	0,11	-0,31	-0,38	-2,24	0,15	-0,18	-0,31	-0,31
7	-5,85	-1,99	-0,59	-0,96	-0,98	3,91	0,41	0,51	0,69	0,69	-0,46	-0,47	-0,24	-0,07	-0,15	-1,14	-0,83	-0,28	-0,37	-0,36	-1,33	-0,84	-0,23	-0,39	-0,48
